# Subtypes of Aggressive Behavior in Children with Autism in the Context of Emotion Recognition, Hostile Attribution Bias, and Dysfunctional Emotion Regulation

**DOI:** 10.1007/s10803-021-05387-w

**Published:** 2021-12-20

**Authors:** Simone Kirst, Katharina Bögl, Verena Loraine Gross, Robert Diehm, Luise Poustka, Isabel Dziobek

**Affiliations:** 1 Clinical Psychology of Social Interaction, Berlin School of Mind and Brain, Department of Psychology, Humboldt-Universität zu Berlin, Berlin, Germany; 2 Berlin, Germany; 3 Department of Child and Adolescent Psychiatry and Psychotherapy, University Medicine, Georg-August Universität Göttingen, Göttingen, Germany

**Keywords:** Autism, Children, Aggression subtypes, Hostile attribution bias, Emotion regulation, Emotion recognition

## Abstract

The causes of aggressive behavior in children with autism are poorly understood, which limits treatment options. Therefore, this study used behavioral testing and parent reports of 60 children with autism to investigate the interplay of emotion misinterpretation and hostile attribution bias in the prediction of different aggressive behaviors. Further, the additional impact of dysfunctional emotion regulation was examined. Path analyses indicated that hostile attribution bias increased verbal and covert aggression but not physical aggression and bullying. Dysfunctional emotion regulation had an additional impact on bullying, verbal aggression, and covert aggression. Emotion recognition was positively associated with hostile attribution bias. These findings provide a first insight into a complex interplay of socio-emotional variables; longitudinal studies are needed to examine causal relationships.

## Introduction

Beyond the core symptoms of autism spectrum conditions (ASC), diverse comorbid behavioral symptoms can hinder the accomplishment of important developmental milestones in children with the diagnosis, with challenging and aggressive behaviors being particularly impactful and limiting (Sullivan et al., 2019). These behaviors considerably restrict school education and treatment, reduce opportunities for interpersonal relationships, and cause feelings of social isolation and stigmatization in parents (Hodgetts et al., 2013). Since 35–50% of children in the autism spectrum show comorbid aggression (Farmer & Aman, 2011; Mazurek et al., 2013), and with aggression being one of the strongest predictors of parental stress (Baker et al., 2002; Hodgetts et al., 2013), it is one of the key factors for seeking treatment (Robb, 2010). It is thus crucial to gain a better understanding of predictors of aggression in children with autism to provide effective prevention and intervention with positive outcomes (Samson, et al., 2015a, 2015b). However, possible causes and correlates are still poorly understood (Hill et al., 2014). Neither autism-related factors (e.g., ASC symptom severity, adaptive behavior) nor autism-unrelated factors (e.g., low IQ, harsh parental practices; Kanne & Mazurek, 2011; Sullivan et al., 2019) seem to be strong and consistent explanatory factors.

Deficits in socio-emotional functions such as diminished empathy (Euler et al., 2017; Pouw et al., 2013), reduced emotion knowledge (Trentacosta & Fine, 2010), and dysfunctional emotion regulation (Röll et al., 2012) have frequently been linked to aggressive behavior in typically developed (TD) children. Dysfunctional emotion regulation was primarily associated with spontaneous reactions to a real or perceived threat (Kaartinen et al., 2014) without any identifiable goal (Blair, 2016) resulting from anger, frustration, or provocation (Crick & Dodge, 1996). Deficits in emotion regulation (e.g., using maladaptive emotion regulation strategies such as rumination or shutting down; Samson et al., 2014) are highly prevalent in children with autism and may result in anger or anxiety being experienced more intensively and frequently than in TD children (Mazefsky et al., 2013; Samson, et al., 2015a, 2015b). In turn, these intensive emotions can cause aggressive behaviors (Bos et al., 2018; Samson, et al., 2015a, 2015b), especially in social situations (Laurent & Rubin, 2004). Additionally, social cognition impairments such as inaccurate interpretations of social intent were found to promote aggressive behaviors (Politte et al., 2018). Thus, it seems plausible that both impaired social-cognitive abilities and emotional functions might explain aggressive behaviors in autistic children.

Even though there is quality research providing empirical support for individual risk factors and predictors of aggression in children with autism, it largely lacks integration, which hinders the effective understanding, prevention, and treatment of aggressive behavior (Chester & Langdon, 2016). The present work is based on the multifaceted *Social Cognitive Information-Processing models* (SCIP models; Crick & Dodge, 1994, 1996; Huesmann, 1998; Lemerise & Arsenio, 2000) because they are to date the most influential and comprehensive frameworks, which are most widely applied to explain aggressive behavior (see reviews Fontaine, 2008; Larkin et al., 2013; Smeijers et al., 2020). When trying to understand the psychosocial sources of aggression in TD children, the SCIP models have proven to be of very good use (van Nieuwenhuijzen et al., 2004) for “developing an integrated picture of how different factors interact and culminate in aggression” (Smeijers et al., 2020).

In the classical version of the SCIP models (Crick & Dodge, 1994, 1996; Huesmann, 1998), aggressive behavior is understood as a consequence of incorrect or biased information processing, especially in social situations. The models posit that the interaction of environmental socializers (e.g., exposure to aggressive models; see as well Bandura, 1973), biological predispositions (e.g., anger proneness), and situational instigators (e.g., provocation) activate an aggression-supporting cognitive style. This style refers to a tendency to interpret situations or the intentions and behavior of others as hostile, even when there is conflicting, missing, or ambiguous information (Guy et al., 2017) and to construct and evaluate aggressive responses as adequate reactions (Görtz-Dorten & Döpfner, 2010). We will hereafter refer to this construct as the “hostile attribution bias”. In aggression-provoking situations, hostile attribution bias can lead to the selection of aggressive responses and thus provoke the development of a stable pattern of aggressive behavior (Musher-Eizenman et al., 2004). The crucial role of hostile attribution bias in the development and maintenance of aggression in TD children has been supported by several investigations (see Martinelli et al., 2018; Verhoef et al., 2019 for recent meta-analyses).

**Fig. 1:**
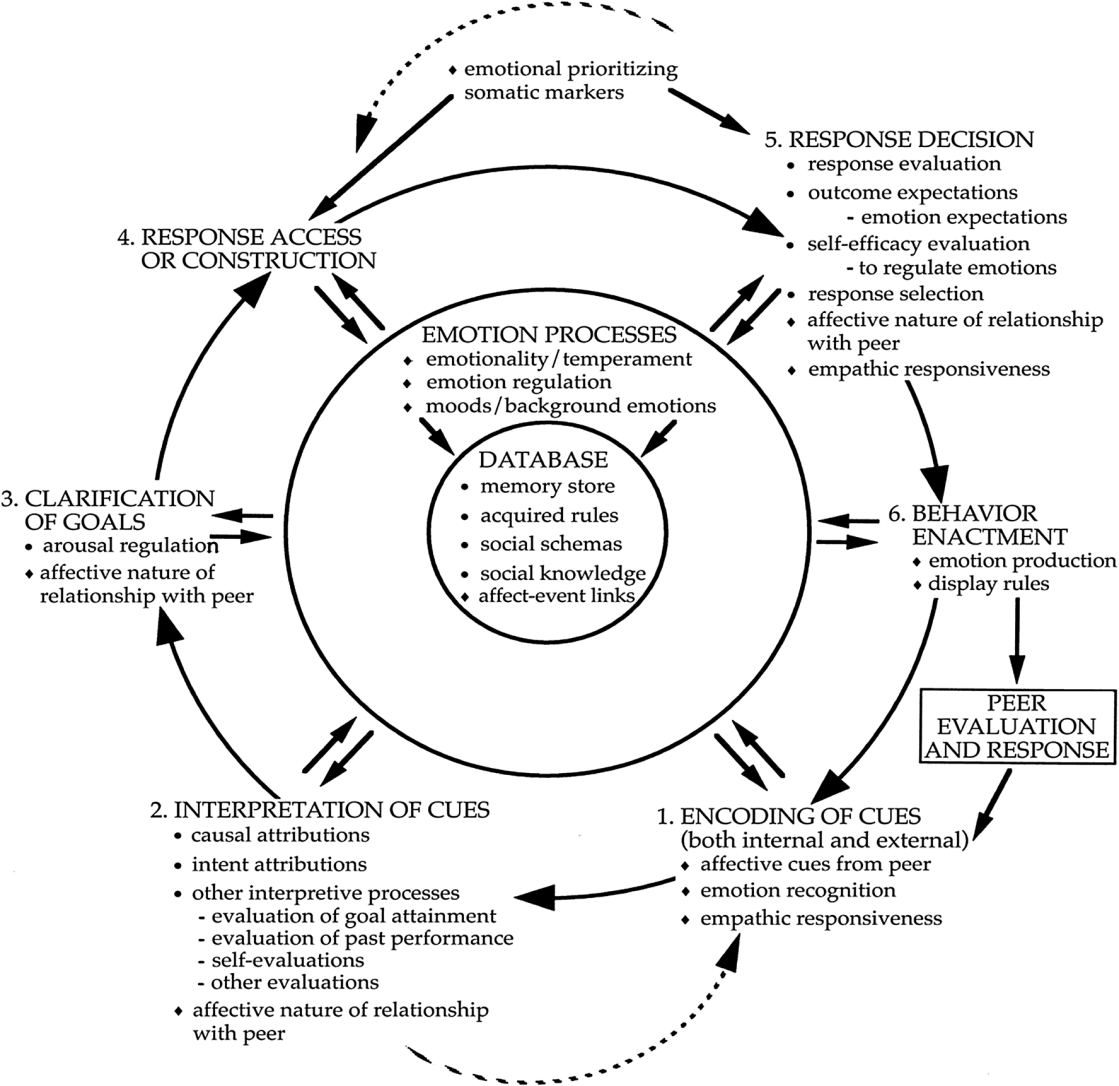
The revised Social Cognitive Information-Processing model (Lemerise & Arsenio, 2000). In this model, aggression is understood as a function of biased social information processing on six mental operations, which are processed rapidly with numerous feedback loops in response to socially challenging situations. Individuals encode incoming information, interpret this information within the particular social context resulting in causal/intent attributions, clarify goals for the interaction, search for possible responses, evaluate possible outcomes for these responses, and then select a response for enactment (van Nieuwenhuijzen et al., 2004). The mental operations are influenced by a database of memorized experiences, acquired rules, social schemas, social knowledge (Crick & Dodge, 1994, 1996), and affect-event links (Lemerise & Arsenio, 2000). Original Figure by Crick and Dodge (1994), *Psychological Bulletin*, 115, p. 74, adapted by Lemerise and Arsenio (2000, p. 113). Copyright © 1994, American Psychological Association. Reprinted with permission

Lemerise and Arsenio (2000) proposed a revised version of the classical SCIP model by including emotion processes (e.g., emotionality/temperament, emotion regulation, and moods, hereinafter: *emotion model,* depicted in Fig. 1). According to this revised model, dysfunctional emotion regulation causes intensive negative experiences of aversive emotions (e.g., anger, anxiety) and general lability, which further enhance and maintain hostile attribution biases and influence later SCIP operations (problem identification and solution, goal clarification, response selection; Helmsen et al., 2012). Empirically, the studies reviewed by Smeijers et al., 2020) tentatively suggest that emotional functions such as emotion recognition and emotion regulation may have distinct influences at different stages of SIP, all having direct or indirect relations to aggressive responses.

Although the SCIP framework promises to be useful in strengthening the theoretical foundations of research on aggression in children with ASC (Ziv et al., 2014), it is rarely applied. Current evidence in children with autism points to difficulties in all SCIP operations when being compared to TD children, including a diminished capacity to efficiently encode socio-emotional information and the existence of hostile attribution biases (Embregts & van Nieuwenhuijzen, 2009; Flood et al., 2011; Mazza et al., 2017; Meyer et al., 2006; Ziv et al., 2014). Ziv et al. (2014) associated hostile attribution bias with a higher frequency of externalizing behaviors.

However, similar to research in TD (see Fontaine, 2008; Smeijers et al., 2020), the integration of cognition and emotion in the understanding of social information processing in aggression is rarely focused on, and studies that investigate whether hostile attribution bias mediates the relationship between deficient emotion processing and aggressive behavior in autism are currently lacking.

### The Present Study

Based on the assumption that a hostile attribution bias mediates the relation between misinterpretation of emotional expressions and aggressive behavior (*classical model*; Crick & Dodge, 1994, 1996), the present study aimed at identifying the additional impact of dysfunctional emotion regulation expressed as lability and negativity in children with autism spectrum conditions. In reference to the modified SCIP models by Lemerise and Arsensio (2000), we hypothesized (H1) that lability-negativity would predict hostile attribution bias and also would have a direct, and positive impact on the presence of aggressive behaviors (*emotion model*; compare Fig. 1). In alignment with Farmer and colleagues (2009, 2016), we view aggression as a multifaceted phenomenon expressed by different subtypes of aggression, which potentially have different responses to treatment and prognoses (Connor et al., 1998). We, therefore, aimed to explore interrelated predictors in physical acts of aggression and more complex forms (verbal aggression and covert aggression, see Table 1 for examples) separately in preschool and primary school children with autism and normal intellectual functioning (IQ ≥ 70). Finally, due to the importance of facial expressions as a modality of social judgment (Frith, 2009) and social-cognitive information processing, we used diminished facial emotion recognition as a potential predictor of hostile attribution bias (compare Lemerise & Arsenio, 2000; Russo-Ponsaran et al., 2015).

**Table 1:** Examples of items of the selected FAVK subscale as a measure of hostile attribution bias, ERC subscale lability-negativity, and C-SHARP subscales measuring subtypes of aggressive behavior

Instrument: subscale	Item
FAVK: Disorder of Social-Cognitive Information Processing *Here: Hostile attribution bias*	If someone steps on his/her foot, he/she insinuates malicious intent. Feels annoyed or provoked by others when they look at him/her funny in his/her opinion. Thinks that many people do not like him/her and have a hostile attitude towards him/her. Often feels unfairly treated.
ERC: Lability-Negativity *Here: Dysfunctional emotion regulation*	Is easily frustrated. Is easily prone to angry outbursts/tantrums. Displays flat affect (expression is vacant and inexpressive; the child seems emotionally absent).
C-SHARP: Verbal Aggression	Calls others insulting names in their absence. Calls others insulting names to their faces. Says ‘‘I hate [someone]’’ or other hurtful things.
C-SHARP: Bullying	Breaks others’ things. Throws objects at others. Crowds others (invades their personal space).
C-SHARP: Covert Aggression	Sneers, ‘‘makes faces’’ at others. Tickles or otherwise physically teases others, even after being asked to stop. If caught, denies having behaved badly.
C-SHARP: Physical Aggression	Bites others. Pulls others’ hair. Pinches others.

*C-SHARP* Children’s scale of hostility and aggression: Reactive/Proactive, *ERC* Emotion Regulation Checklist, *FAVK* German Inventory of Aggressive Behavior in Children, Subscale: Disorder of Social-Cognitive Information Processing

The present study was part of a registered six-week multicenter, randomized, pragmatic clinical trial testing a tablet-based intervention in children with autism (Kirst et al., 2020; DRKS-ID: DRKS00009337; Universal Trial Number (UTN): U1111-1175–5451). Since no TD children participated in the trial, no comparison group was available for the current study.

## Methods

### Participants

Out of 184 screened children with ASC, 82 children were eligible for the RCT trial, from which 60 children (50 males) between 5.0 and 10.11 years (*M* = 8.0 years, *SD* = 1.6) fulfilled the sample inclusion criteria. A power analysis revealed that this sample size is sufficient to detect an expected effect size of hostile attribution bias on children’s aggressive behavior of Cohen’s *d* = 0.33 (see meta-analysis by Verhoef et al., 2019) with 80% power (1- β) at a two-sided 5% α level and emotion regulation as an additional predictor. The inclusion criteria were (1) complete testing data in predictor variables (emotion recognition, emotion dysregulation, hostile attribution bias), (2) intellectual functioning within the normal range (IQ ≥ 70) as assessed by a composite score of the Raven's Colored Progressive Matrices intelligence test (2002) and by the Peabody Picture Vocabulary Test, 4th revision (Dunn & Dunn, 2015), and (3) a clinical consensus ICD-10 (WHO, 1994) diagnosis of childhood autism, Asperger syndrome, atypical autism or pervasive developmental disorder not otherwise specified (PDD-NOS). Diagnosis was established by specialized and experienced multi-professional teams using a variety of measures and clinical judgment. Results of the Autism Diagnostic Observation Schedule (ADOS-G/ADOS-2; Lord et al., 2000, 2015; Merkle et al., 2016) were provided by caregivers or clinicians for 53 participants, who were eligible for the present study. To confirm the ASC diagnosis, the Autism Diagnostic Interview-Revised (ADI-R) short version (Hoffmann et al., 2015) was administered to all participants, and autism symptomatology was further assessed by using the Social Communication Questionnaire (Rutter et al., 2006). Interfering neurological/medical conditions (except for well-treated epilepsy) were ruled out by parental report. The subscale “Aggressive Behavior” of the Child Behavior Checklist (CBCL 4/18; German version; Döpfner & Arbeitsgruppe Deutsche Child Behavior Checklist, 2003) was used to assess clinical severity by age-group comparisons according to gender. Additionally, the frequency of aggressive and auto-aggressive behaviors ranging from 1: “never” to 4: “several times a week” was rated by parents in an unstandardized report.

### Procedure

The children were assessed at three study centers. These were based at Humboldt–Universität zu Berlin, Germany (HU) and at two University Departments of Child and Adolescent Psychiatry and Psychotherapy with specialized outpatient clinics for children/adolescents with ASC in Augsburg (KJPP AUG), Germany, and Vienna (MedUni Wien), Austria. Additional participants were recruited through autism care units and parent organizations in Germany and Austria, as well as through a study website (www.zirkus-empathico.de). The assessment of the data reported here took place before the main intervention of the RCT. The questionnaires for parents were provided online on the SoSci-Survey platform (Leiner, 2014) and Lime Survey (Limesurvey GmbH, 2016). The RCT trial received ethical approval from the Ethics Committee at HU (2015/10/07) and the clinical authorities in the two outpatient clinics. Written informed consent was obtained from the children’s legal guardians after receiving a detailed study description. Families received €7/hour as compensation.

### Measures

#### Facial Emotion Recognition

We tested facial emotion recognition accuracy by using a series of 28 pictures of facial affect by Ekman and Friesen (1976). Pictures were presented on a computer screen and participants had to choose the correct emotion label out of a wordlist of six basic emotional states (happiness, sadness, fear, disgust, anger, surprise), intermixed with the word “neutral”. Labels were displayed in random order. Each correctly identified emotion label scored one point, and the total sum comprised the accuracy score of the participant. Children with sufficient reading skills (7-10y) read by themselves. For younger/non-literate children, labels were read aloud and keys were pressed by the testing operator according to the child’s verbal answer. Analyses with 73 children of the total RCT sample with valid data at baseline and 64 additionally measured TD children revealed good reliability of the Ekman & Friesen picture set (McDonald’s Omega = 0.97).

#### Emotion Dysregulation: Lability-Negativity

Dysfunctional emotion regulation was assessed by the “lability-negativity” subscale of the 15-item Emotion Regulation Checklist (ERC; Shields & Cicchetti, 1997), which measures lack of flexibility, anger dysregulation, and mood lability on a four-point rating scale (1: “never”; 2: “sometimes”; 3: “often”; 4: “almost always”). The ERC is a parent questionnaire, which is suitable for children aged 6–12 years. The second subscale (“emotion regulation”, 8 items), which targets the expression of emotions, empathy, and constructive emotional self-awareness was not included in the present study because it was shown to be more strongly correlated with functional social skills, while the lability-negativity subscale was positively associated with hyperactive, externalizing, and internalizing behavior (Henriques Reis et al., 2016). The ERC shows good convergent validity with similar instruments and an adequate internal consistency (LabNeg: α = 0.96; ER: α = 0.83; Shields & Cicchetti, 1997).

#### Hostile Attribution Bias

The subscale “Disturbances in social information processing” (see Table 1) of the German Inventory of Aggressive Behavior in Children (FAVK; Görtz-Dorten & Döpfner, 2010) was used to assess hostile attribution bias.

The scale targets aggression-promoting attitudes, thought patterns, and response tendencies towards others as summarized under the concept of an aggression-supporting cognitive style by the SCIP models (Görtz-Dorten & Döpfner, 2010) in children between 4–14 years. It is rated separately with regard to aggressive tendencies (a) towards peers, and (b) towards adults on a four-point-rating scale ranging from 0: “not at all true” to 3: “definitely true.” Ratings are subsequently summed up to two total scores with higher scores corresponding to more severe dysfunction. For the present study, we used the parent-report form and calculated a mean score of the peer and adult subscales to collapse both scores into one. The FAVK showed satisfactory internal consistency in non-referred samples as well as good discriminative validity and high internal consistency in a clinical sample (Cronbach’s α = 0.95; Benesch et al., 2013; Görtz-Dorten & Döpfner, 2010).

#### Subtypes of Aggressive Behavior

The parent questionnaire C-SHARP (“Children’s scale of hostility and aggression: Reactive/Proactive”; Farmer & Aman, 2009, 2010) records aggressive behaviors and hostility in children with developmental disorders (such as ADHD and ASC) in 48 items (short–version) on five subscales: verbal aggression, bullying, covert aggression, physical aggression, and hostility (Table 1). In the current study, the hostility subscale was excluded from analyses because its items are similar to those of the ERC lability-negativity subscale (e.g., reacts suddenly or impulsively to minor provocations; shouts at others in anger). Each item of the C-SHARP is rated on a problem and a provocation dimension. The problem dimension assesses the frequency and severity of aggressive behavior in the last month on a scale ranging from 0: “does not occur” to 3: “severe and/or frequent problem”. Higher sum scores describe more severe behaviors in the respective aggression subscale. The reliability and validity of the five problem scales of the English original version were shown to be sufficient, and the coefficient alpha ranged from moderate (0.74, physical aggression) to high (0.92, verbal aggression). Behaviors that were classified as present in the problem scale (≥ 1) were rated on the provocation dimension as either being a response to external circumstances (provoked; reactive, score: -2 to -1), or as being a planned action (not-provoked; proactive; score: + 1 to + 2), with zero being neutral. Following Farmer et al. (2015), the provocation scores were summed up for each subscale and categorized into one out of three categories: “reactive” (sum less than zero), “neutral” (sum of zero; similar rates of reactive and proactive behavior), or “proactive” (sum greater than zero). The internal consistency for this approach was acceptable (verbal aggression: α = 0.81, bullying: α = 0.81, covert aggression: α = 0.72, physical aggression: α = 0.68; Farmer et al., 2015). For the current study, the English original of the questionnaire was translated into German and back-translated into English in cooperation with the authors of the questionnaire. Cronbach’s alpha for the four problem scales was good for verbal aggression (α = 0.90), bullying (α = 0.85), and covert aggression (α = 0.83), but not for physical aggression (α = 0.65).

### Statistical Analyses

Pearson’s correlation analyses were calculated to examine the links between aggression subtypes and demographic/clinical characteristics (age, nonverbal/verbal IQ, autism social symptoms (SCQ)). All statistical tests were two-tailed and were conducted pairwise. The Bonferroni–Holm procedure was applied to correct significance thresholds to account for the accumulation of *type I error* due to multiple comparisons. Reports include corrected significance values (*p*), and *r* statistics for Pearson’s *r*.

**Fig. 2:**
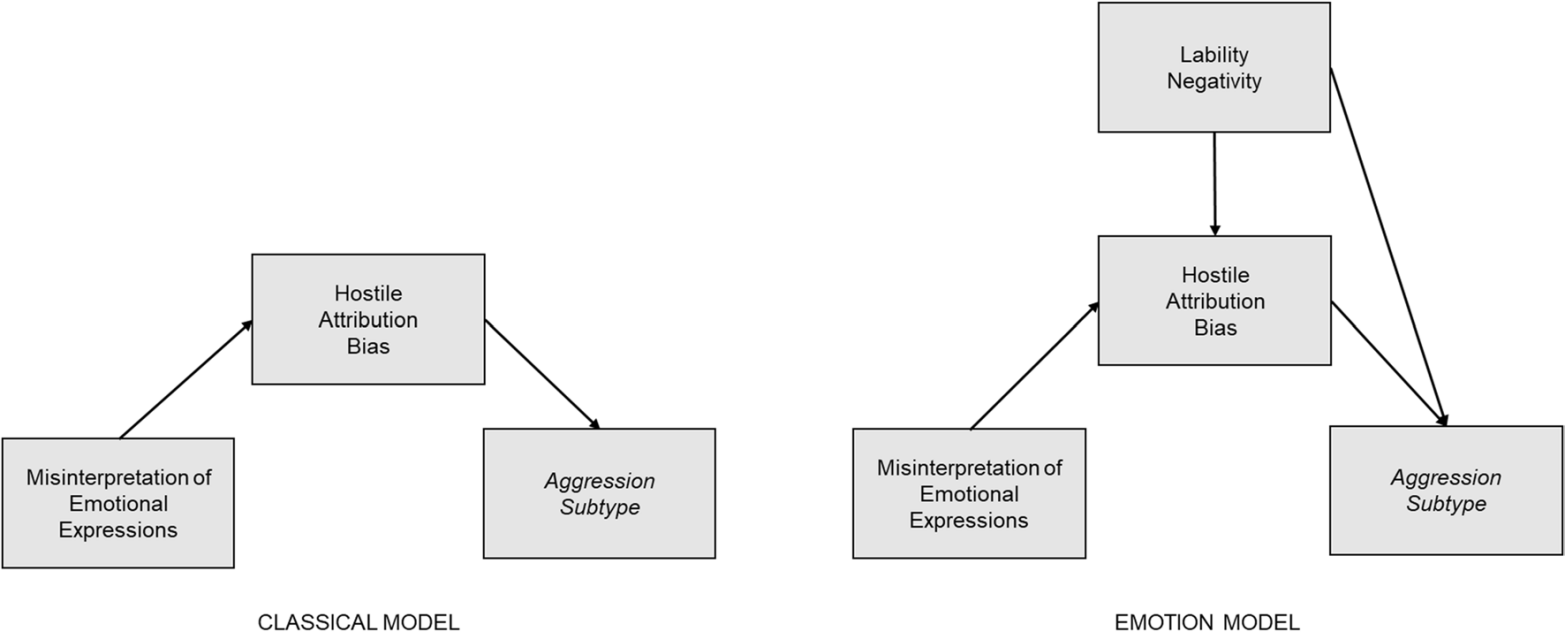
Paths models testing the *emotion model* (H1; compare Lemerise & Arsenio, 2000) against the *classical model* (compare Crick & Dodge, 1994, 1996; Huesmann, 1998)

As proposed in our hypotheses, and in reference to the modified SCIP models (Lemerise & Arsenio, 2000; Fig. 2), we specified a path model including dysfunctional emotion processes (*emotion model*), which was compared with the classical version of the SCIP models (*classical model;* Crick & Dodge, 1994, 1996). The model comparison was used to evaluate if the more complex *emotion model* explains aggressive behaviors better than the *classical model*, which only relies on cognitive processes such as emotion recognition and hostile attribution bias. In our specific case, we specified the *classical model* by facial emotion recognition accuracy predicting hostile attribution bias (as assessed through the FAVK score), which in turn predicts different aggression subtypes (physical aggression, bullying, verbal aggression, and covert aggression as measured by the C-SHARP). The *emotion model* includes dysfunctional emotion regulation (as assessed by the ERC subscale lability-negativity) as an additional predictor of hostile attribution bias and aggression subtype with hostile attribution bias mediating the relationship between lability-negativity and aggression. Both models were specified through maximum likelihood estimation with robust standard errors and a Satorra-Bentler scaled test statistic. The MLM estimator was used because multivariate normality could not be assumed for every model. Model fit was validated by using model fit indices (comparative fit index, *CFI*, root-mean-square-error of approximation, *RMSEA*, and standardized root-mean-square residual, *SRMR*). The *emotion model* was compared to the *classical model* based on *CFI* comparisons (*CFI classical model* minus *CFI emotion model*) with negative delta *CFI* pointing to a better fit of the *emotion model*; the cut-off for a meaningful difference was set to -0.002 (Meade et al., 2008). Additionally, a sample-size adjusted Bayesian information criterion (Adj. *BIC*) and Akaike information criterion (*AIC*) were used for model comparison, with smaller values indicating better model fit (Merkle et al., 2016). All comparisons were run separately for each form of aggressive behavior to explore the predictive value of the two proposed models for the different subtypes of aggression. Significance thresholds were corrected by applying the Bonferroni–Holm procedure to account for the accumulation of *type I error* due to multiple comparisons. Reports include corrected significance values (*p*). All analyses were performed in R (Version 1.3.1073, R Core Team, 2018).

## Results

### Sample Characteristics

The demographic and clinical characteristics of n = 60 children are displayed in Table 2.

**Table 2:** Demographics and clinical characteristics of the total sample, N = 60

Variable	*M*	*SD*	*Range*
Age (years)	8.0	1.5	5.3–10.8
CPM, Nonverbal IQ	105.8	19.1	67.0–135.0
PPVT, Verbal IQ	101.7	17.7	65.5–134.5
ADOS-G, Total (N = 45)	11.7	4.0	4.0–20.0
ADOS-2, Total (N = 8)	10.8	2.9	8.0–15.0
SCQ, Total Score	21.2	6.5	3.0–35.0
ADI-R short	5.9	5.9	2.0–8.0
	N	%	
Males	50	83.3	
*ASC Diagnosis (ICD-10)*			
Childhood Autism	8	13.3	
Asperger Syndrome	34	56.7	
Atypical Autism	4	6.7	
PDD-NOS	14	23.3	
*Comorbidity*			
None/Unknown	45	75.0	
ADHD/ADD	10	16.7	
Epilepsy	1	1.7	
Other	4	6.7	
CBCL Subscale Aggressive Behavior: Above clinical cut-off (T ≥ 70)	33	55.0	
*Parent Report, Frequency of Aggression*			
Never	13	22.8	
Infrequent	9	15.8	
Several times per month	14	24.6	
Several times per week	21	36.8	
*C-SHARP Provocation Scale: Verbal Aggression*			
Reactive	34	28.1	
Reactive-Proactive	5	9.4	
Proactive	7	59.4	
No verbal aggression	4	3.1	
*C-SHARP Provocation Scale: Bullying*			
Reactive	32	65.3	
Reactive-proactive	10	20.4	
Proactive	7	14.3	
No bullying	0	0.0	
*C-SHARP Provocation Scale: Covert Aggression*			
Reactive	26	52.0	
Reactive-proactive	6	12.0	
Proactive	16	32.0	
No covert aggression	2	4.0	
*C-SHARP Provocation Scale: Physical Aggression*			
Reactive	19	35.0	
Reactive-proactive	10	18.9	
Proactive	7	13.2	
No physical aggression	17	32.1	

*ADHD/ADD* Attention Deficit Disorder, *ADI-R short* Autism Diagnostic Interview-Revised short version, *ADOS-G* Autism Diagnostic Observation Scale Generic, overall total (communication + reciprocal social interaction), *ADOS-2* Autism Diagnostic Observation Scale-2, overall total (social affect + restricted and repetitive behavior), *ASC* Autism Spectrum Conditions, *CBCL* Child Behavior Checklist, *CPM* Colored Progressive Matrices, *C-SHARP* Children’s scale of hostility and aggression: Reactive/Proactive, *PPVT* Peabody Picture Vocabulary Test, *PDD_NOS* Pervasive developmental disorder not otherwise specified, *SCQ* Social Communication Questionnaire

FAVK data was available for 60 participants, and 55 parents rated their child on the C-SHARP aggression assessment. The cut-off (T > 70) for clinically significant aggression on the CBCL subscale was met by 55% of the total sample (n = 33) with the majority (62%) showing aggressive behaviors several times a month (25%), or several times a week (37%). The most prevalent subtypes were covert aggression (*M* = 9.0, *SD* = 5.4), bullying (*M* = 8.9, *SD* = 6.8), and verbal aggression (*M* = 8.7, *SD* = 7.8), while physical aggression showed the lowest prevalence (*M* = 2.2, *SD* = 2.5). There was no significant difference between boys and girls for all forms of aggressive behavior (verbal aggression: *t*(54) = 0.99, *p* = 0.341; bullying: *t*(54) = 0.35, *p* = 0.730; covert aggression: *t*(54) = 0.62, *p* = 0.547, physical aggression: *t*(53) = 1.49, *p* = 0.154). Analysis revealed that children were more likely to engage in reactive than proactive aggression as reflected by the C- SHARP provocation dimension (Table 2).

### Correlation Analyses

After correcting for multiple comparisons, neither age nor nonverbal or verbal IQ or autism social symptomatology (SCQ) correlated significantly with the aggression subtypes or the predictor variables (emotion recognition, hostile attribution bias, lability-negativity) (Table 3).

**Table 3:** Correlation matrix (Pearson’s *r*) between demographic variables (age, verbal and nonverbal IQ, autism social symptomatology), predictors (emotion recognition, lability-negativity, hostile attribution bias), and aggression subtypes

	Age	CPM	PPVT	SCQ	EmoRec	LabNeg	HAB	VerbAggr	Bully	CovAggr
Age										
CPM	− .18									
PPVT	− .02	**.47								
SCQ	.27	− .21	− .12							
EmoRec	.35	.33	***.54	− .12						
LabNeg	.03	− .13	.08	.26	− .01					
HAB	.23	.08	.26	.02	.31	***.53				
VerbAggr	.20	.03	.08	.18	.01	***.59	***.70			
Bully	− .17	.13	.24	− .02	..08	***.59	***.59	***.65		
CovAggr	.19	.10	.21	.18	.16	***.67	***.67	***.80	***.76	
PhysAggr	− .11	.29	.28	.15	.13	.39	.39	.41	***.72	***.54

Significance thresholds were corrected for multiple comparisons by using the Bonferroni–Holm procedure

*Bully* bullying, *CovAggr* covert aggression, *CPM* Colored Progressive Matrices (nonverbal IQ), *EmoRec* emotion recognition, *HAB* hostile attribution bias, *LabNeg* Lability-Negativity, *PhysAggr* physical aggression, *PPVT* Peabody Picture Vocabulary Test (verbal IQ), *SCQ* Social Communication Questionnaire (autism social symptomatology), *VerbAggr* verbal aggression

^*^p < .05, **p < .01, *** *p* < .001

### Path Analyses

Path analyses revealed an acceptable to a good fit for the *classical model* in all aggression subtypes (Table 4). In favor of H1, model comparisons revealed a better model fit for the *emotion model* when compared to the *classical model* as indicated through *CFI*, *AIC*, and *BIC* values. This means that the predictive power of the models was enhanced when lability-negativity was included as a second predictor of hostile attribution bias and the respective aggression subtypes.

**Table 4:** Model fit indices (CFI, AIC/Adj. BIC) for the classical model and the emotion model

	Classical Model			Emotion Model			
	*CFI*	*AIC*	Adj. *BIC*	*CFI*	*AIC*	Adj. *BIC*	*∆ CFI*
VerbAggr	.95	676.7	672.1	1.00	651.9	645.1	-.05
Bullying	1.00	676.8	672.2	1.00	648.2	642.4	.00
CovAggr	1.00	629.3	624.7	1.00	596.9	590.1	.00
PhysAggr	1.00	570.1	565.5	1.00	550.8	543.8	.00

Model comparison was done by subtracting fit indices (CFI) of the *emotion model* from those of the *classical model* with negative indices indicating a better fit. Lower AIC and adjusted BIC indicate a better fit of the respective model

*AIC* Akaike information criterion, *Adj. BIC* sample-size adjusted Bayesian information criterion, *CFI* comparative fit index, *CovAggr* covert aggression, *HAB* hostile attribution bias, *PhysAggr* physical aggression

**Fig. 3:**
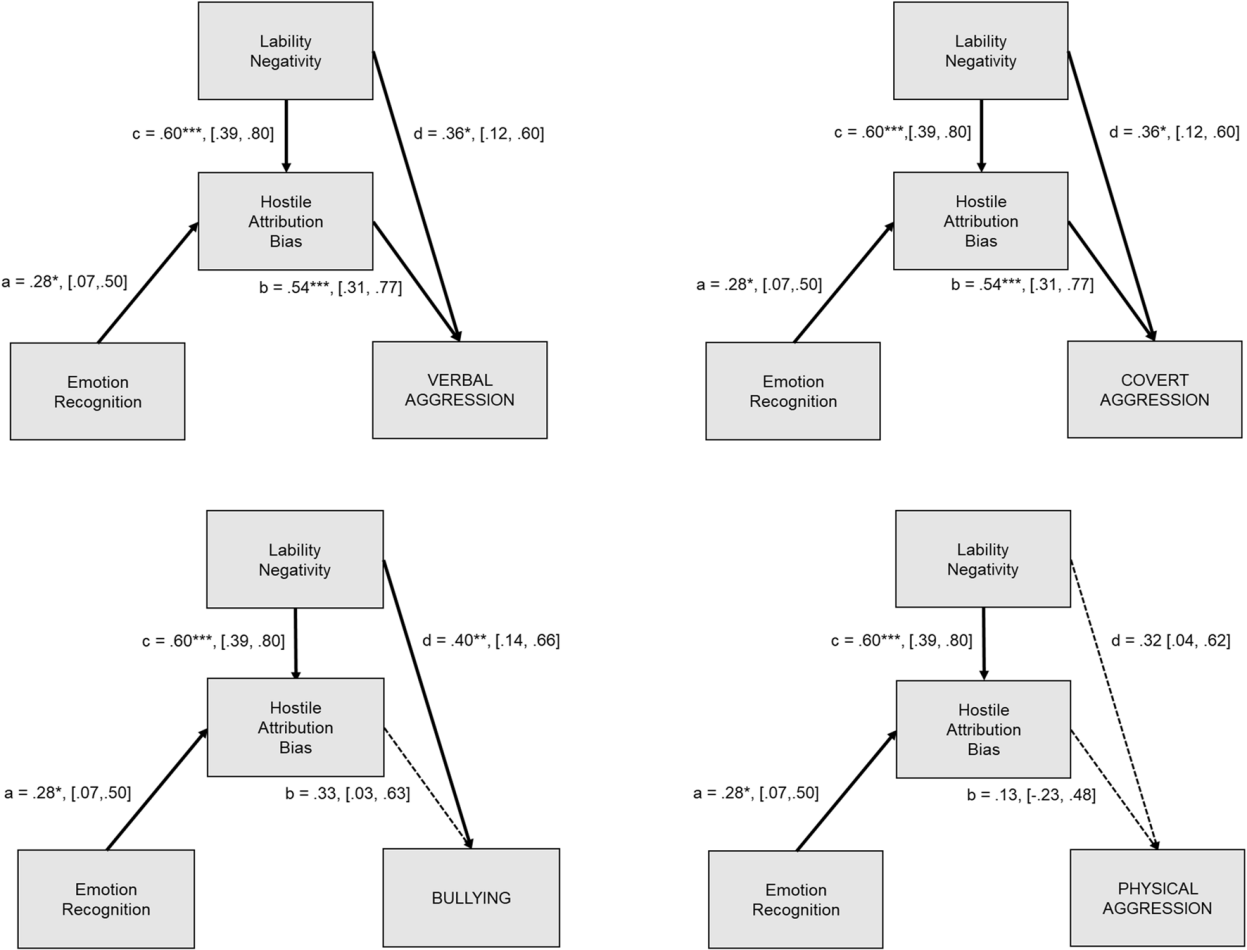
Results of the *emotion model*: Regression coefficients (*β*s) from path models depicting **a** the direct effect of emotion recognition on hostile attribution bias, **b** the direct effect of hostile attribution bias on the respective aggression subtype, **c** the direct effect of lability-negativity on hostile attribution bias, and **d** the direct effect of lability-negativity on aggressive behavior subtype. Standardized estimates and confidence intervals are displayed in parenthesis. *p < 0.05, **p < 0.01, *** *p* < 0.001 (Bonferroni–Holm corrected)

By applying the *emotion model*, and after correcting for multiple comparisons (Table 4, Fig. 3), we found hostile attribution bias being positively predicted by emotion recognition accuracy (standardized estimates with confidence intervals, *b* = 0.283, *p* = 0.032, [0.074, 0.496]) and lability-negativity (*b* = 0.594*, p* < 0.001, [0.390, 0.797]). Hostile attribution bias was a significant positive predictor of verbal aggression (*b* = 0.545, *p* < 0.001, [0.375, 0.715]) and covert aggression (*b* = 0.540, *p* < 0.001, [0.308, 0.772]), but not of bullying (*b* = 0.332*, p* = 0.124, [0.030, 0.634]) and physical aggression (*b* = 0.126, *p* = *1*.00, [-0.227, 0.478]). Lability-negativity had a direct positive effect on verbal aggression (*b* = 0.272, *p* = 0.004, [0.113, 0.430]), bullying (*b* = 0.403*, p* = 0.008, [0.143, 0.662]), and covert aggression (*b* = 0.356, *p* = 0.016, [0.116, 0.596]), but not on physical aggression (*b* = 0.321, *p* = *0.1*08, [0.036, 0.607]). Hostile attribution bias partly mediated the relationship between lability-negativity and verbal, or respectively, covert aggression.

## Discussion

We aimed gaining a better understanding of the socio-emotional sources of aggressive behaviors, and specifically the impact of dysfunctional emotion regulation, in pre-and primary school children with autism, hoping to inform the development of customized interventions for target groups and their families. We based our approach on the theoretical considerations of the classical version of the SCIP models (Crick & Dodge, 1994, 1996; Huesmann, 1998) in comparison to its revised version (Lemerise & Arsenio, 2000; here: *emotion model*). Hence, we hypothesized in reference to the classical model, that misinterpretation of emotional expressions would predict hostile attribution bias, which in turn, should enhance different aggressive behaviors as measured via the C-SHARP parent questionnaire. In addition – and by referring to the emotion model –, we hypothesized that parent-rated lability-negativity, as a symptom of dysfunctional emotion regulation, should have an additional impact on hostile attribution bias and different aggressive behaviors*.*

Our results were multifaceted. First, emotion recognition accuracy predicted the tendency to attribute hostile intent *positively* in all four subtypes of aggression. Second, for all four subtypes of aggression, we found the more complex emotion model including lability-negativity, to describe our data better than the classical model without lability-negativity. Third, lability-negativity was directly related to all aggression subtypes, *except* for physical aggression. Further, the positive impact of lability-negativity on aggressive behavior was *partly* mediated by hostile attribution bias in the case of verbal and covert aggression, while hostile attribution bias did not affect physical aggression and bullying. Overall, our results confirm our hypothesis for verbal and covert aggression: lability-negativity had a direct influence on the respective aggression subtype as well as an indirect influence via hostile attribution bias. Due to the absent effect of hostile attribution bias on bullying, the hypothesis was not fully confirmed here. No confirmation was found for the influence of lability-negativity and hostile attribution bias on physical aggression. Nevertheless, these findings underline a complex interplay between hostile attribution bias and emotion dysregulation, which differently affects aggression subtypes. In the following, we interpret these findings in the context of past aggression research in children with TD and with ASC.

### The Impact of Emotion Recognition on Hostile Attribution Bias

According to the SCIP, the first step of social-cognitive information-processing is the correct encoding and processing of others’ emotions to make moral judgments (Chester & Langdon, 2016; Lemerise & Arsenio, 2000). Previous studies identified positive relationships between emotion recognition and later SCIP steps (e.g., problem identification and solution, goal clarification, response selection) in TD children with and without mild intellectual impairments (Bauminger et al., 2005; Meyer et al., 2006; Schultz et al., 2004; van Nieuwenhuijzen & Vriens, 2012) and children with autism (Russo-Ponsaran et al., 2015). In line with this, a lack of cognitive empathy (understanding of others' emotions; Dziobek et al., 2008) was related to higher aggressive tendencies in some studies with TD children (Euler et al., 2017; Mayberry & Espelage, 2007). Hence, it was rather unexpected that children with *better* emotion recognition skills were rated as having a *more pronounced* tendency to attribute hostile intent to others in our autistic sample. Interestingly, Pouw et al. (2013) reported a positive relationship between self-rated cognitive understanding of others’ emotions and aggression levels in children with autism but not TD children. They argued that emotional content of any kind, such as when correctly understanding the emotions of others, could activate empathic arousal, which is perceived as aversive (personal distress) due to dysfunctional emotion regulation and therefore triggers aggressive behaviors. However, from a longitudinal perspective, it seems plausible, that the causal relationship between the variables is reversed: a child might have developed a tendency to attribute hostile intent in the first place due to frequent negative social experiences (e.g., being teased, or being excluded because of autism-related social impairments; see Ziv et al., 2014 for further causes). As a consequence, the child might have trained emotion recognition skills/cognitive empathy more intensively to detect potentially hostile or aggressive cues early (compare hypervigilance to hostile cues, Helmsen et al., 2012) to prevent negative experiences. Indeed, Embregts and van Nieuwenhuijzen (2009) found boys with autism and mild intellectual impairment to strongly focus on negative and emotional information in video-presented vignettes of social situations. Longitudinal studies are therefore needed to further disentangle the complex relationship between emotion understanding and aggression (compare Quan et al., 2019).

### The Interplay of Hostile Attribution Bias and Lability-Negativity

We observed the proposed interplay between dysfunctional emotion regulation and hostile attribution bias for verbal aggression (e.g., saying hurtful things, insulting others) and covert aggression (e.g., physically teasing others against their will, sneering at others). Thus, the revised SCIP models (here: *emotion model*, Lemerise & Arsenio, 2000) seem to be a valid approach for explaining these more complex aggression subtypes in children with autism. Interestingly, due to the missing impact of hostile attribution bias on bullying and physical aggression in our sample, the revised SCIP models might not be informative for the more physically or overtly expressed aggression subtypes. This is surprising given studies demonstrating significant impairments in SCIP operations in children with autism when compared with TD children including hostile intent attribution in ambiguous situations (e.g., Flood et al., 2011; Russo-Ponsaran et al., 2015; Ziv et al., 2014), and studies demonstrating relations between hostile intent attribution and aggressive behavior in TD children (Martinelli et al., 2018; Verhoef et al., 2019). However, Helmsen et al. (2012) reported no association between hostile intent attribution and aggression in TD children. A study by Coy et al. (2001) further found that preschool boys with oppositional defiant disorder were no more likely to attribute hostile intentions in ambiguous situations than boys of the control group. Finally, bullying, which is defined as malicious actions to strategically harm another person in order to gain or preserve power or reputation (Volk et al., 2017), is thought to arise from deficiencies, or persistent biases in the early stages of the SCIP (Crick & Dodge, 1999). In contrast to this view, current studies (e.g., Guy et al., 2017) do not support that TD bullies make more hostile attributions in response to ambiguous social information, which would indicate biases in early SCIP operations.

These mixed results might in part be due to differences in methodology such as different measurements of emotion processes, hostile attribution bias, and aggressive behavior (see Helmsen et al., 2012). Furthermore, it may be relevant to operationalize hostile attribution bias analog to the aggression subtype in focus. In their meta-analysis, Martinelli et al. (2018) found *physically* aggressive TD children to attribute hostile intent especially in response to *physically provocative* situations (e.g., when being hit with a ball). In contrast, children engaging in *relational* aggression (infliction of harm via actual or threatened damage to, or control of, relationships; Crick & Grotpeter, 1995) primarily displayed *relational* hostile attribution bias (e.g., in response to vignettes targeting ambiguous social situations like not being invited to a friend’s birthday). The items of the FAVK subscale that were used here (e.g., thinks that many people do not like him/her and have a hostile attitude towards him/her; often feels unfairly treated) seem to address hostile intent attributions, which are more closely associated with complex aggression subtypes such as verbal or covert aggression than with physical aggression. Since physical hostile attribution bias was not specifically targeted here, the assumption that a tendency to attribute hostile intent might have an impact on the relationship between lability-negativity and physical aggression in children with autism should be reevaluated with a broader set of hostile attribution bias items.

Furthermore, our results for bullying, with lability-negativity having an impact on this subtype while hostile attribution bias does not, underline the interpretation of the C-SHARP bullying subscale by its authors Farmer and Aman (2010, 2011). Based on their findings in children with autism, they suggested that the items of the bullying subscale (e.g., throwing objects at others, invading personal space) might *not* represent malicious actions intended to harm other persons in this population, but rather impulsive, socially inadequate responses to stressful environmental conditions. More plastically, the “children engage in physical ‘communication’ when frustrated” (Farmer & Aman, 2010, p.278) because they are incapable of alternative actions (Mazza et al., 2017) due to autism-related social skills impairments (e.g., difficulties to communicate desires, or personal needs in adequate ways). Therefore, we could potentially conclude that our results for the bullying subscale might *generally* account for simple physical acts of aggression towards others, with lability-negativity being a prominent predictor. Additional predictors related to social interaction and communication impairments potentially having an impact on later SCIP operations (e.g., response access/construction; response decision, see Fig. 2) should be investigated in future research.

Surprisingly, the C-SHARP subscale, which explicitly targets physical aggression, was not associated with lability-negativity. Besides its questionable reliability in our sample (Cronbach’s Alpha = 0.65; but 0.74 in Farmer & Aman, 2010), it might be that the low physical aggression rates (M = 2.18, SD = 0.33), with 17 children (32%) showing no physical aggression at all, resulted in low variance and therefore insufficient statistical power to detect the proposed relations in the rather small sample (n = 54). These low physical aggression rates might be due to a low representation of children with intellectual impairment, limited language ability, and low adaptive functioning; factors which are associated with an increased risk for aggressive behavior for individuals with autism (Hill et al., 2014; Mazefsky et al., 2013). Farmer et al. (2015) found physical aggression being related to lower IQ levels in autistic children, while more complex aggression subtypes (verbal/covert aggression) were associated with higher IQ, better adaptive behavior, and older age. However, we did not observe correlations between demographic/clinical variables (autism symptom severity, age, verbal/nonverbal IQ) and the aggression subtypes in our sample, which shows a relatively narrow age range and (high) IQ level when compared to Farmer et al. (2015). We might conclude that, especially the more physically expressed subtypes (here: bullying and physical aggression), should be targeted with carefully designed longitudinal studies to disentangle a range of different potential predictors (dysfunctional emotion regulation, hostile attribution bias, lack of social skills, etc.) in larger samples under the theoretical perspective of the SCIP models.

### Limitations

By using a cross-sectional mediation approach, the developmental trajectories and directionality of the relations between risk factors (here: emotion recognition, hostile attribution bias, lability-negativity) leading to aggressive behaviors cannot be disentangled sufficiently to fully understand causal relationships. According to Cole and Maxwell (2003, 2007) mediation consists of causal processes that unfold over time. Thus, using cross-sectional approaches to mediation typically generate substantially biased estimates of longitudinal parameters (Maxwell & Cole, 2007). As pointed out by Helmsen et al. (2012), it is most likely that the relationship between emotion regulation, social information processing, and aggressive behavior is bidirectional. Therefore, longitudinal studies are needed to investigate the causal direction of these relationships. Second, we cannot rule out that observer biases confounded relationships between the different constructs. However, we had to largely rely on parent questionnaires due to the young age of the children. We encourage future studies to use more objective measures to assess emotion regulation abilities and hostile attribution bias (e.g., pictorial interviews using vignettes, compare Helmsen et al., 2012; Mazza et al., 2017; Ziv et al., 2014). Lastly, we have not included a typically developed comparison group, given that we relied on data from an RCT including only children with autism. Thus, we cannot make inferences about the specificity of the reported results. However, much is known about factors predicting aggressive behavior in TD, which we sought to supplement with insights from autism in the current work as a preliminary step. Nevertheless, future research should compare autistic to TD children to investigate between-group differences in the pattern of the interplay of these socio-emotional predictors of aggression.

### Implications

Even though our understanding is still limited, the results reported here may have implications for designing and selecting targeted interventions for children with autism and comorbid aggression as well as for future research on the topic. First, our study showed the important role of emotion regulation for verbal and covert aggression as well as for bullying. Thus, emotion regulation competencies (e.g., awareness of own emotions, impulse/anger control, functional emotion regulation strategies) should be given priority in therapy (compare Helmsen et al., 2012; or novel technology-based approaches like “Zirkus Empathico”; Kirst et al., 2020). This might also strengthen autistic children to better deal with negative arousal potentially induced by others’ emotional displays (Kliemann et al., 2013; Pouw et al., 2013), which could, in turn, enable more fruitful training of understanding others’ socio-emotional cues. Given our results, emotion regulation competencies may also diminish hostile attribution biases, and thus exert additional beneficial effects on the reduction of externalizing behavior via this indirect route. Since particularly emotionally engaging social situations were found to elicit the automatic and emotional processes that activate hostile attribution bias, interventions should assess and target biases in similar and naturalistic situations (Verhoef et al., 2019). Additionally, the specific pattern of aggressive behavior in children with autism should be carefully identified for each patient to allow individualized interventions. Beyond assessing the most prevalent aggression subtypes and their function for the individual, the nature of hostile attribution biases should be examined to allow customized and effective interventions. Behaviors summarized by the bullying and potentially by the physical subscale might be effectively reduced by strengthening social skills in addition to emotion regulation strategies, while more complex aggression subtypes such as verbal and covert aggression could be targeted by identifying and modifying aggression-promoting attitudes, thought patterns, and response tendencies towards others through cognitive-behavioral approaches. Indeed, interventions modifying SCIP in TD children (e.g., Hudley & Graham, 1993; Lochman & Wells, 2002) have been proven relatively effective (Kazdin, 2003).

Finally, our behavioral findings of the interplay between hostile attribution bias, emotion regulation, and abnormal behavior should be further investigated from different perspectives (e.g., socio-cognitive, developmental, neurobiological) in autism samples. So far, a prominent role of emotion dysregulation and related personal traits such as impulsivity in moderating the relationship between social cognition and aggression has been demonstrated cross-sectionally (e.g., Musher-Eizenman et al., 2004) and longitudinally in TD individuals. For example, Blandon et al. (2010) and Halligan et al. (2013) reported a causal role for problematic regulation of negative emotions at age one and, respectively, two, and the etiology of externalizing psychopathology at age five, and seven. By using a longitudinal approach in a large adolescent sample (N = 585), Fite et al. (2008) found impulsivity moderating the relationship between cognitions (here: positive endorsement of aggressive responses in hypothetical, ambiguous situations) at age 11–13 and aggressive behavior at age 14–17. Interestingly, only moderately to highly impulsive individuals showed a significant association between aggression-prone cognitions and aggressive behavior. Likewise, Goldweber et al. (2011) suggested that individual differences in executive functions (here: inhibiting behavior, shifting attention, and controlling emotions) may account for stability in aggressive social information processing (SIP). They found children aged 7–13 years with a stable aggressive SIP pattern exhibiting more executive functions problems than children who showed a decline in aggressive SIP over one year.

From a neurobiological perspective, the medial prefrontal cortex (mPFC) as the “cortical control board” (Xu et al., 2019, page 2), has been found to play an essential role for emotion regulation and, among others, for sociability (Xu et al., 2019). In addition to autism, abnormal activity in the mPFC has been shown for other psychiatric disorders (e.g., depression, anxiety, schizophrenia, addiction; see review by Xu et al., 2019). Identifying the specific pattern of cortical activation in response to emotion regulation processes inherent to autism may help to differentiate between autism and potentially co-occurring psychiatric disorders (e.g., depression, anxiety). Furthermore, localizing distinct cortical areas in the mPFC related to autism-specific deficits in emotion regulation processes (compare findings for major depression, Rive et al., 2013) and studying their connections to regions involved in higher-order socio-cognitive processing may result in a more in-depth understanding of the interrelated abnormalities underlying aggressive behavior in some individuals with autism.

## Conclusion

Taken together, the revised SCIP models (Lemerise & Arsenio, 2000) seem to be a promising approach for investigating various risk factors and their interplay for aggressive behaviors in children with autism. It demonstrated a prominent role of dysfunctional emotion regulation in causing different aggression subtypes, which might be differently affected by a tendency to attribute hostile intent to others. By applying the model, future studies with bigger samples, control groups, and longitudinal designs should identify distinct patterns of aggressive behaviors by investigating the interplay of various socio-emotional predictors in children with autism.
